# Antibody glycosylation in COVID-19

**DOI:** 10.1007/s10719-022-10044-0

**Published:** 2022-01-29

**Authors:** Tamas Pongracz, Gestur Vidarsson, Manfred Wuhrer

**Affiliations:** 1 Center for Proteomics and Metabolomics, Leiden University Medical Center, Leiden, The Netherlands; 2 Department of Experimental Immunohematology, Sanquin Research, and Landsteiner Laboratory, UMC, University of Amsterdam, AmsterdamAmsterdam, Netherlands

**Keywords:** IgG glycosylation, Antibody glycosylation, SARS-CoV-2, COVID-19, Glycomics, biomarker

## Abstract

Antibody glycosylation has received considerable attention in coronavirus disease 2019 (COVID-19) infections and recently also in vaccination. Antibody glycosylation and in particular immunoglobulin G1 fucosylation levels influence effector functions and are therefore key parameters for assessing the efficacy and safety of severe acute respiratory syndrome coronavirus 2 (SARS-CoV-2) directed immune responses. This review article summarizes and interprets recent research into antibody glycosylation in COVID-19. Experimental approaches for analyzing the glycosylation of SARS-CoV-2-directed antibody responses are evaluated. The pronounced dynamics, effector functions, clinical utility, and regulation of antibody glycosylation in COVID-19 are assessed. Future research on the role of antibody glycosylation in COVID may cover the glycosylation of other antibody classes beyond immunoglobulin G, the regulation of antibody glycosylation, and the role of non-canonical antibody receptors in determining effector functions.

**Graphical abstract:**

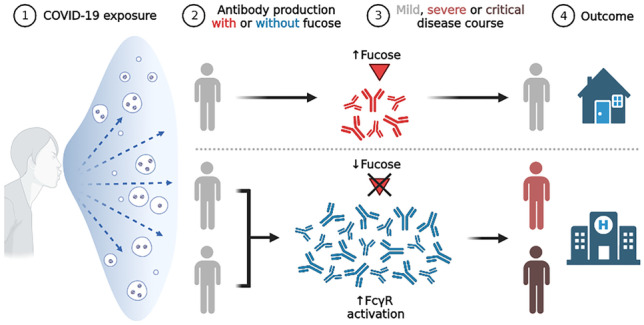

## Introduction

Antibodies are abundant soluble glycoproteins in the circulation, various biofluids and mucosal layers playing essential roles in the adaptive immune response [1]. Beyond antigen binding and neutralization via the fragment antigen-binding (Fab) portion, their immune-regulatory role lays in steering diverse effector functions via their fragment crystallizable (Fc) portion [1–3]. With their wide-spanning functions including antigen binding and neutralization, opsonization, mediating complement-dependent cytotoxicity (CDC) as well as antibody-dependent cellular cytotoxicity and phagocytosis (ADCC and ADCP, respectively), antibodies are front-line elements in host defense against infectious agents [3]. Immunoglobulin G (IgG) is the most abundant antibody in plasma and is comprised of four isotypes [4]. The Fc tails of IgG are co- and post-translationally modified by glycosylation. The resulting *N*-glycan is an important structural component that fine-tunes effector functions [2]. Notably, adaptive diversification of this Fc-linked *N*-glycan may elicit qualitatively different immune responses by varying their potential to activate complement and by altering their binding to Fc receptors present on a range of immune cells [5].

During homeostasis hardly any intra-individual variation is observed in the composition of the plasma-derived total (or bulk) IgG glycome [5–7]. With various physiological and pathological changes, such as aging, pregnancy, hormonal adjustments, and inflammatory and metabolic diseases, the IgG glycome is changing. Likewise, IgG glycosylation associates with body mass index (BMI) and smoking. In addition, IgG glycosylation is influenced by genetic and epigenetic determinants [5, 8].

Substantial alterations of IgG glycosylation are concomitant with various infectious diseases and vaccinations against those [5, 9–11]. These glycosylation alterations have mostly been studied on circulatory total IgG manifesting themselves systemically in an acute or chronic manner. However, these total IgG glycosylation changes may also partially be driven by buildup of skewed glycosylation of antigen-specific IgG [5, 8–14]. Fucose-deficient pathogen-specific IgG has recently been identified as a general initial glyco-phenotypic response characteristic of viral infections such as human immunodeficiency virus (HIV), Dengue, and severe acute respiratory syndrome coronavirus 2 (SARS-CoV-2) – all being enveloped viruses that bud through cell membranes [15–18]. Similar afucosylated IgG has also been seen in antigen-specific responses to other foreign membrane antigens such as platelet and red blood cell alloantigens in pregnancy [19–21] and *Plasmodium falciparium* antigens on red blood cells [16]. Antibody fucosylation is of paramount importance, because the lack of core fucose amplifies affinity of IgG to its cognate Fcγ receptors (FcγR) thereby escalating ADCC [2, 22].

Recent studies have pointed towards associations between IgG1 glycosylation – especially fucosylation – and coronavirus disease 2019 (COVID-19) severity, but study results are not directly comparable due to differences in cohorts, disease phases and methodologies. This encouraged us to concisely review the available evidence in order to identify commonalities and address discrepancies in methodologies and patient cohorts. Eventually, we provide a broader outlook on IgG glycosylation patterns in SARS-CoV-2 messenger ribonucleic acid (mRNA) vaccination and provide perspectives on the utility of antigen-specific IgG glycosylation analysis as a factor in assessing efficacy and safety of both pathogen- and vaccine-induced immune responses.

## Antibodies and COVID-19

SARS-CoV-2 infections show largely diverse disease courses, and it became evident during the ongoing pandemic, that an evoked robust anti-SARS-CoV-2 immune response, commonly considered as protective, can in fact lead to aggravated immunopathologies [23, 24]. Disease worsening in COVID-19 has been observed to be concurrent with seroconversion and activity of the adaptive immune system with IgG playing a major role [24, 25]. For this adverse reaction excessive FcγR activation by IgG antibodies seems to be instrumental [15, 26–28]. Interestingly, since the early stages of the COVID-19 pandemic, it has been recognized that while some individuals develop life-threatening conditions, others control the infection with relatively mild symptoms [24]. Demographic factors and comorbidities are two of the predisposing factors of disease course [29], still, there is an urgent need for additional determinants and early biomarkers with higher specificity in predicting outcomes.

## Methods for the assessment of antibody glycosylation in COVID-19

An early study by Petrovic et al. applied ultrahigh-performance liquid chromatography with fluorescence detection (UPLC-FLD) for analyzing total *N*-glycans of IgG, covering both Fc and Fab glycans alike of all IgG subclasses. This method may be considered the gold standard for antibody glycosylation analysis and features a particularly high precision. Petrovic et al. focused on total IgG glycosylation analysis, and no analysis of SARS-CoV-2 spike protein (S) specific antibodies or anti-SARS-CoV-2 receptor binding domain (RBD) antibodies was pursued [30].

**Fig. 1:**
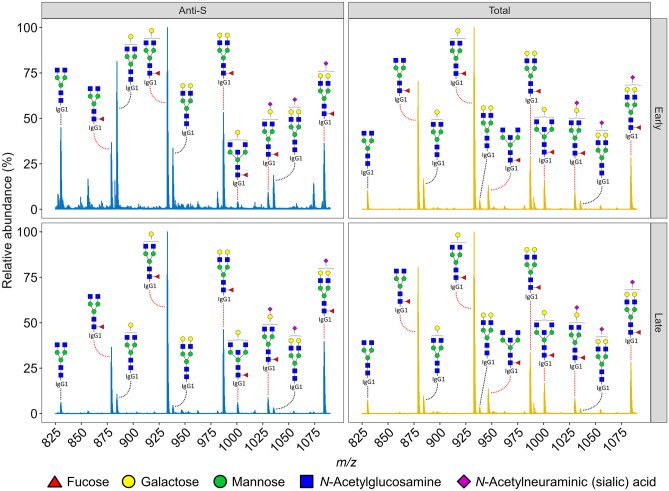
Representative MS spectra of anti-S (left) and total (right) IgG1 glycopeptides of a hospitalized COVID-19 patient at an early (top) and a late (bottom) timepoint. The early timepoint illustrates the glycosylation pattern at 14 days after symptom onset (around the time of seroconversion), while the late timepoint illustrates the glycosylation pattern 14 days later. Dotted lines indicate fucosylated (red line) and afucosylated (black line) glycoforms. All annotated glycopeptide species are triple protonated. Structural annotations are based on manual spectral interpretation and literature [5]

In the studies of Larsen et al., Hoepel et al., Bye et al. and Pongracz et al., a common, liquid chromatography – mass spectrometry (LC–MS)-based method was employed to characterize total, anti-S, and to a limited extent anti-SARS-CoV-2 nucleocapsid (N) IgG glycosylation following their affinity purification [15, 26, 27, 31]. This approach builds on microtitration plate-based adsorption of antibodies to viral proteins using a modification of enzyme-linked immunosorbent assay (ELISA) antibody detection methods. Tryptic Fc glycopeptide analysis is achieved using an LC–MS high-throughput bottom-up proteomics workflow followed by targeted data extraction and label-free quantification. The major advantage of this method besides its robustness is that it gives site-specific information, thereby providing a focus on Fc glycosylation while readily distinguishing IgG subclasses (Fig. 1) [32].

Similarly to the afore approach, Chakraborty et al. used LC–MS for the bottom-up analysis of IgG Fc glycosylation, albeit using multiple-reaction monitoring (MRM; on a triple quadrupole MS) for the target detection and quantification of pre-selected glycopeptides [28]. Farkash et al. detected glycopeptides by parallel reaction monitoring (PRM) on Orbitrap MS [33].

An alternative MS-based method potentially useful for the assessment of IgG Fc glycosylation in COVID-19 is middle-up antibody analysis, as presented for anti-RBD IgG in a study by Melani et al. Even though this method is promising, the observed high complexity due to multiple different amino acid sequences and glycan structural heterogeneity complicates Fc glycosylation analysis using this approach [34].

Conversely to MS-based methods, Ankerhold et al. used a lectin-based assay to characterize anti-S and anti-N IgG fucosylation. The principle of this method lies in the preferential binding of the lectin *Aleuria aurantia* to α1,6-fucose linked to *N*-acetylglucosamine, which has been exploited to quantify corresponding fucosylation levels in an ELISA-setting [35].

The comparability of the results of the various assays has for a big part not been established. In particular, it would be of interest to see how the results of the MRM- based and PRM-based MS methods relate to established UPLC-FLD and LC–MS high-throughput glycomics approaches. For the latter two methods, very good comparability has been demonstrated for applications in a biomedical setting, providing a basis for the integration of data obtained with these methods [36].

## Dynamics and potential clinical utility of IgG Fc glycosylation in COVID-19

High-throughput technological advances allowed to routinely analyze the IgG glycomes in large clinical cohorts, which contributed to our understanding on how IgG glycosylation signatures associated with changes in health and disease [5, 37]. Recently, multiple related findings suggested that altered S protein-specific or receptor binding domain (RBD)-specific IgG1 Fc glycosylation is a promising candidate severity marker in COVID-19 (Table 1) [15, 26, 28, 31, 35].

**Table 1:** Overview of studies of SARS-CoV2 antigen-specific antibody glycosylation in COVID-19

**Study**	**Setting**	**Study population**	**Antigen used for affinity-capturing**	**Method to analyse IgG glycosylation**	**Functional or other assays used**	**Longitudinal sampling**	**Glycosylation pattern**
Larsen et al. [15]	Disease	1) non-ARDS (outpatient) 2) ARDS (inpatient)	spike, nucleocapsid	LC–MS	1) Cytokine release assay 2) Antibody levels	Yes	transient
Chakrabortky et al. [28]	Disease	1) non-ICU (inpatient) 2) ICU (inpatient) 3) outpatient 4) pediatric (past infection)	receptor binding domain	LC–MS (MRM)	1) FcγRIIIA bindig 2) NK cell degranulation assay 3) Monocyte stimulation and cytokine measurement	Yes, but improperly defined	long-lasting
Hoepel et al. [26]	Disease	1) ICU (inpatient) 2) SARS-CoV-2 negative individuals 3) SARS-CoV-2 positive but spike protein negative individuals; Samples used herein were a subpopulation of baseline samples used by Larsen et al. [15]	spike	LC–MS	1) Cytokine release assay 2) Antibody levels 3) RNAseq 4) Endothelial barrier function 5) Platelet adhesion 6) Drug inhibition assessment	-	-
Bye et al. [27]	Glycoengineered monoclonal antibody-based study			LC–MS	1) Thrombus formation assay 2) Drug inhibition assessment	-	-
Ankerhold et al. [35]	Disease	1) severe (inpatient) 2) critical (inpatient)	spike, receptor binding domain, nucleocapsid	Lectin-based assay	1) FcγRIIIa activation assay; 2) Neutralization assay	No	-
Pongracz et al. [31]	Disease	1) non-ICU (inpatient) 2) ICU (inpatient)	spike	LC–MS	1) Cyto- and chemokine levels (Luminex) 2) Antibody levels	Yes	transient
Farkash et al. [33]	Vaccination and disease	1) vaccinees 2) convalescent 3) mild (inpatient) 4) severe (inpatient)	spike	LC–MS (PRM)	1) Antibody levels 2) FcγRIIIa binding 3) C1q binding	Yes	transient

IgG1 Fc fucosylation is in the spotlight of most these studies, and high levels of afucosylated anti-S or anti-RBD IgG1 have been construed as a motif associated with exacerbated immunopathologies in COVID-19 [15, 26, 28, 31, 35]. Larsen et al. [15] and Chakraborty et al. [28] demonstrated proinflammatory, low-fucosylation signatures of anti-S and anti-RBD IgG1 in patients with severe respiratory complications, respectively. Intensive care unit (ICU)-admitted patients who developed acute respiratory distress syndrome (ARDS) were found to show lower anti-S IgG1 fucosylation as compared to outpatients with mild symptoms [15]. Chakraborty et al. looked at inpatients at both the ICU and non-ICU as well as outpatients and asymptomatic pediatrics, and found anti-RBD IgG1 fucosylation to be lower in the hospitalized groups as compared to the outpatients and pediatrics [28], which is largely in-line with the findings of Larsen et al. [15].

Of note, anti-S IgG1 fucosylation could not further discriminate between the various hospitalized groups [28], which is in line with observations of Ankerhold et al. [35] and Pongracz et al. [31]. Specifically, the latter study found no differences in anti-S IgG1 fucosylation levels in patients stratified for different disease severities or ICU admission. Ankerhold et al. argue that circulating multimeric immune complexes – not monomeric IgG – potentially enriched in low-fucosylated IgG drive immunopathology in COVID-19 [35]. The study was performed a lectin-based assay to quantify anti-S, anti-RBD and anti-N IgG fucosylation levels, in contrast to the LC–MS based methods used by the other groups (Table 1), with the lectin assay not differentiating IgG isotypes. Also, the antigen-specificity of the IgGs in the immune complexes remained undetermined, therefore the suggested involvement of low-fucosylated anti-S IgG in the described immune complex formation would need further confirmation [35].

Next to fucosylation, also galactosylation, sialylation and bisection of anti-S IgG1 showed pronounced dynamics in COVID-19, with initially high levels of galactosylation and sialylation as well as low levels of bisection [31]. After hospitalization, anti-S IgG1 galactosylation, sialylation and bisection became more similar to total IgG1 glycosylation, with galactosylation and sialylation dropping, whilst bisection was increasing [31]. Anti-S IgG1 bisection, galactosylation and sialylation correlated with disease severity as well as a broad range of inflammatory and clinical parameters [31].

Bisection of IgG was found to be low in severe COVID-19, as shown both for total IgG [30] and anti-S IgG1 [15]. Another study likewise showed low anti-S bisection relative to total IgG1, but intriguingly, bisection was positively associated with ICU admission, disease severity and survival [31], unlike in a similar study, where bisection on anti-RBD IgG1 did not show discriminative potential between ICU and non-ICU patients [28]. Remarkably, a pronounced skewing of bisection, galactosylation and sialylation of anti-S IgG1 as compared to bulk IgG1 glycosylation was observed for COVID-19 patients that did not need to be admitted to ICU [31]. Conversely, a very limited skewing of these IgG1 glycosylation traits characterized the ICU patients. Of note, these glycosylation differences were already apparent with hospitalization, evincing their potential as promising severity marker in COVID-19. Further studies are needed, also assessing anti-S IgG1 glycosylation in patients prior to hospitalization to establish the prognostic value of these signatures regarding the development of disease severity and the need of different treatment regimens [31].

Some of the above studies found remarkably dynamic glycosylation patterns as exemplified for one patient in Fig. 1. Intriguingly, the pronounced dynamics characterizing anti-S fucosylation [15, 31] were found to be extremely stable in previous studies assessing fucosylation levels to various antigens in alloimmune and infectious diseases, persisting over a decade [16, 20, 38, 39]. Transient dynamics of the anti-S glycosylation were likewise found with respect to bisection, galactosylation and sialylation. Although the observed dynamics in these studies were also found for total IgG1 – albeit to a lesser extent – it is unknown whether total IgG1 glycosylation changes are largely caused by anti-S and other SARS-CoV-2-specific antibody neo-production with skewed glycosylation – or whether antibodies of other specificities are contributing [15, 31]. On the contrary, in the study of Chakraborty et al. a long-lasting, unchanged glycosylation pattern was observed for anti-RBD antibodies, which may be due to later sampling [28], as the afucosylated IgG was only observed in the first week after seroconversion [15]. Of note, the comparability of anti-S and anti-RBD glycosylation profiles has not been demonstrated yet, and antigen-specific differences in the IgG1 fucosylation patterns could therefore explain part of the different dynamics. Interestingly, anti-S and anti-RBD antibodies showed differential performance in *in vitro* functional assays [40], providing an incentive to sort out possible differential Fc modification and receptor engagement of these SARS-CoV-2-directed antibody subpopulations.

## Regulation of IgG Fc glycosylation

IgG glycosylation is influenced by demographic factors such as age and sex [5]. Furthermore, IgG glycosylation is influenced by (epi)genetic factors, pregnancy, hormones, menopause, lifestyle factors such as smoking and BMI, and environmental factors [5]. On the cellular level, IgG glycosylation is considered to be largely determined during biosynthesis in plasma cells, with key factors being the expression levels of glycosyltransferases, Golgi topology and pH, availability of monosaccharides, protein production kinetics and transport mechanisms [8]. Infectious disease-associated shifts occurring on pathogen-specific antibodies have been broadly described, suggesting a controlled modulation of the immune response by adjusted IgG glycosylation, as summarized elsewhere [5, 8, 11].

In SARS-CoV-2, it has been hypothesized that the antigen context gives rise to IgG afucosylation, with host membrane-associated antigen presentation as a pre-requisite for the induction of low-fucose responses. This led to the postulation of a signal at the viral protein-displaying host plasma membrane that would trigger afucosylated IgG responses in B cells, yet the nature of this signal remains elusive [15]. Furthermore, age and sex have been associated not only with total, but also with anti-S and anti-RBD IgG1 bisection, galactosylation and sialylation, and it has therefore been suggested to account for demographic besides temporal confounders more mindfully in future studies [28, 31]. Overall, the dynamics of anti-S IgG1 glycosylation early on in infection and vaccination may be due to a rapid increase in antibody production, which is paralleled by a rapid increase in antibody concentrations during a phase when a rapid expansion of clonal B cells and formation of new plasma blasts and plasma cells is occurring [15]. An early, largely afucosylated IgG1 response with still relatively low antibody concentrations may within a few days or weeks be followed by a much higher production of largely fucosylated IgG1. While all available evidence suggest that IgG glycosylation is governed by the secretion machinery in B cells, in particular for fucosylation which can be vastly different from the bulk IgG, it has to be stressed that further research is needed to investigate possible post-secretion glycosylation processing. It will be important to identify and characterize SARS-CoV-2 specific B cell populations and their location in lymphoid tissues for developing an understanding of the pronounced dynamics of antibody glycosylation in COVID-19.

## Functional consequences of altered Fc glycosylation

Altered pathogen-specific antibody glycosylation has been reported to impact their inflammatory potential and functionality [3, 5, 11, 41]. For example, persistent accumulation of agalactosylated gp120-specific IgG has been reported in HIV independently from disease state [42]. Similarly, pathogen-directed antibodies in the active phase of *Mycobacterium tuberculosis* infection showed largely agalactosylated glycosylation patterns, unlike in latent tuberculosis [43]. IgG sialylation has been linked to anti-inflammatory activity [44], with the underlying molecular mechanisms remaining obscure. The most well-characterized and understood glycan modification appears to be afucosylation, which directly enhances antibody functionality with increased FcγRIII affinity and elevated ADCC [2, 22]. For example, afucosylated non-neutralizing IgG antibodies were found in Dengue infection showing enhanced binding to FcγRIIIa *in vitro* and triggered platelet reduction *in vivo*. These afucosylated IgG1 were postulated to contribute to the immunopathology via antibody-dependent enhancement (ADE) [17].

Larsen et al. used an *in vitro* cytokine release assay to demonstrate that glycoengineered monoclonal anti-S IgG1 carrying afucosylated glycans – when incorporated in immune complexes with recombinantly expressed spike protein – induced elevated interleukin (IL)-6 release in monocyte-derived macrophages (expressing FcγRIIIa), as compared to its normally fucosylated counterpart. However, it is worth to note that fucosylation levels of the used glycoengineered anti-S IgG1 were way below those observed in patients. Accordingly, the onset of low-fucosylated anti-S IgG1 in critically ill patients was accompanied by a rise in IL-6 (together with C-reactive protein and D-dimer) upon longitudinal sampling [15].

Consistent with these observations, Chakraborty et al. showed that the affinity and dissociation constant of patient sera, and of both isolated patient-derived and glycoengineered anti-RBD IgG1 for recombinant FcγRIIIa was proportional to the degree of IgG1 afucosylation. Additionally, the same study used in vitro stimulation assays to quantify NK cell degranulation as well as cytokine production by primary monocytes. Using both of these assays employing immune complexes formed with patient-derived IgG1 or glycoengineered anti-RBD IgG1 immune complexes, they found that low-fucosylated anti-RBD IgG1 indeed impacted ADCC as exemplified by increased IL-6, tumor necrosis factor and IL-1β production, when compared to normally fucosylated alternatives [28].

Hoepel et al. identified the combination of high titers and low fucosylation of anti-S IgG1 as the potential predisposing factor of severe COVID-19. Using an array of techniques, they likewise found increased cytokine release, a pro-inflammatory ribonucleic acid sequencing profile as well as disrupted endothelial barrier function and platelet adhesion associating with anti-S IgG1 afucosylation. Using the same glycoengineered anti-S IgG1 as Larsen et al., they found the proinflammatory cytokine production by macrophages (including IL-6) was blocked by FcγRIIIa-blocking antibodies (but curiously also with FcγRIIa-blocking antibodies, perhaps suggesting synergism in signaling between these redundant receptor pairs). In addition, this was also inhibited by small molecule drug fostamatinib to counteract afucosylated anti-S IgG1-induced inflammatory responses *in vitro* by blocking ADCC-associated, which is of therapeutic interest in COVID-19 [26].

Bye et al. reported on the association of anti-S IgG1 afucosylation with prothrombotic platelet activation using *in vitro* models and highlighted the role of a platelet-specific Fc receptor FcγRIIa [27]. This finding is remarkable, as the FcγRIIa is not known to prefer afucosylated IgGs [2], and molecular insights as to how afucosylation contributes to prothrombotic platelet activation are lacking. Further research is needed to evaluate the potential contribution of afucosylated IgG responses to thrombotic complication in COVID-19.

Using a cell-based reporter system, Ankerhold et al. showed that serum pools originating from COVID-19 patients right upon hospitalization were potent in FcγRIIIa activation, after normalizing to antigen-specific IgG titers, although the findings were independent of clinical manifestation and could not be used for the differentiation between severe and critical cases [35].

Together, these findings consistently revealed a marked, proinflammatory fucosylation signature on plasma-derived anti-S IgG1 originating from severely ill, SARS-CoV-2 infected inpatients [15, 26, 28, 31, 35]. Most studies additionally provide convincing multi-angle *in vitro* functional evidence associating anti-S IgG1 afucosylation with enhanced immune cell activation [15, 26, 28, 35] or blood clotting abnormalities [27]. Interestingly however, the observed low-fucosylation signatures in hospitalized patients did not show the same associations with cytokines, chemokines and acute phase proteins as shown in *in vitro* assays [31].

## Vaccination

By vaccination with foreign antigens representing parts or even whole attenuated or killed infectious agents, immune responses are evoked, including induction of pathogen-specific neutralizing antibody and long-lived memory B cell production, that are jointly capable of alleviating and/or eliminating the infections during a later encounter. Vaccination provides a highly interesting setting where *in vivo* Fc glycosylation and dynamics thereof can be followed in a relatively well controlled model, given that for example the time between a primer and a booster shot is largely comparable between vaccinees [11]. Hitherto, limited efforts have been made to monitor the pathogen-specific antibody glycosylation repertoire in human [10, 45], even though a well-defined glyco-phenotype has been suggested to be important for vaccine efficacy and safety [11, 41].

A recent study by Farkash et al. investigated anti-RBD antibody glycosylation patterns longitudinally, as elicited by the mRNA vaccine BNT162b2. Dynamic Fc compositions and immune receptor engagement were found, different from those in the setting of a natural infection or in convalescents. These antibodies were characterized by high fucosylation and low bisection [33]. While this study provides interesting insights into vaccine-induced IgG glycosylation responses, it featured relatively low sample numbers and in particular low time resolution (2 weeks between booster and sampling), and further studies are needed to unravel antibody glycosylation dynamics for commonly used mRNA and vector-based COVID-19 vaccines in antigen-naive persons versus those with a (previous) COVID-19 infection.

## Conclusions and future perspectives

Initiation of the adaptive immune response against SARS-CoV-2 is indispensable to fight the infection. It appears that IgG antibodies are key components in protection against COVID-19 with an important role for glycosylation and resulting Fc-mediated effector functions. Recent studies have collectively indicated that a distinct, pro-inflammatory, low-fucosylation glycosylation phenotype marks circulatory IgG produced against the SARS-CoV-2 spike protein in hospitalized patients. This response has been suggested to characterize IgG responses against host-membrane embedded antigens, albeit the underlying mechanisms revealing this await further elucidation. While anti-S IgG1 afucosylation marks high COVID-19 disease severity and associates with numerous inflammatory markers *in vitro*, other glycosylation features including bisection, galactosylation and sialylation show promising associations with disease severity pointing towards their clinical biomarker potential. The regulation of antibody glycosylation is poorly understood, and further research is needed to provide a mechanistic understanding of antibody glycosylation at the cellular and systemic level, to design intervention strategies targeting antibody glycosylation in COVID-19 as well as other diseases.

While the role of IgG1 afucosylation in steering effector functions via FcγRIII interaction is receiving due attention, the role of other glycosylation features such as galactosylation, sialylation and bisection is poorly understood. For anti-SARS-CoV-2 antibody galactosylation and sialylation, further research is needed on its role in effector functions and complement activation [46] and possible contribution to disease pathology. Likewise, antibody glycosylation-dependent effects such as FcγRIIa-mediated prothrombotic platelet activation [27] must be investigated further to comprehend the glycosylation-dependent interaction of antibodies with receptors. In addition, future studies should investigate glycosylation of other clinically relevant immunoglobulin classes such as IgA as well as tissue-specific antibody glycosylation patterns that may reflect the inflammatory state during the disease course more accurately.

In view of the observed glycosylation signatures in COVID-19, we believe that pathogen-specific antibody glycosylation is an important determinant of effector functions, that could be a promising severity marker for infection induced as well as efficacy marker for vaccine induced antibodies in the future.

## Data Availability

Data used on Fig. 1 originated from data generated during a previous study by Pongracz et al., which is available on request from the corresponding author [31].

## Abbreviations

ADCC: Antibody-dependent cellular cytotoxicity
ADCP: Antibody-dependent cellular phagocytosis
ADE: Antibody-dependent enhancement
CDC: Complement-dependent cytotoxicity
COVID-19: Coronavirus disease 2019
ELISA: Enzyme-linked immunosorbent assay
Fab: Fragment antigen-binding
Fc: Fragment crystallizable
FcγR: Fcγ receptors
HIV: Human immunodeficiency virus
ICU: Intensive care unit
IgG: Immunoglobulin G
LC-MS: Liquid chromatography – mass spectrometry
MRM: Multiple reaction monitoring
mRN: Messenger ribonucleic acid
N: Nucleocapsid
PRM: Parallel reaction monitoring
RBD: Receptor binding domain
S: SARS-CoV-2 spike protein
SARS-CoV-2: Severe acute respiratory syndrome coronavirus 2
UPLC-FLD: Ultrahigh-performance liquid chromatography with fluorescence detection
